# Oxidant‐ induced preconditioning: A pharmacologic approach for triggering renal ‘self defense’

**DOI:** 10.14814/phy2.15507

**Published:** 2022-10-28

**Authors:** Richard A. Zager

**Affiliations:** ^1^ Department of Medicine University of Washington Seattle Washington USA; ^2^ Fred Hutchinson Cancer Center Seattle Washington USA

**Keywords:** ferritin, IL‐10, iron sucrose, Nrf2, tin protoporphyrin heme oxygenase 1

## Abstract

Acute kidney injury (AKI) is a common event, occurring in ~5% and ~35% of hospitalized and ICU patients, respectively. The development of AKI portends an increased risk of morbidity, mortality, prolonged hospitalization, and subsequent development of chronic kidney disease (CKD). Given these facts, a multitude of experimental studies have addressed potential methods for inducing AKI prevention in high‐risk patients. However, successful clinical translation of promising experimental data has remained elusive. Over the past decade, our laboratory has focused on developing a method for safely triggering AKI protection by inducing “kidney preconditioning” in mice by the intravenous administration of a combination of Fe sucrose (FeS) + tin protoporphyrin (SnPP). These agents induce mild, but short lived, ‘oxidant stress’ which synergistically activate a number of kidney ‘self‐defense’ pathways (e.g., Nrf2, ferritin, IL‐10). Within 18–24 h of Fe/SnPP administration, marked protection against diverse forms of experimental toxic and ischemic AKI results. FeS/SnPP‐mediated reductions in kidney injury can also indirectly decrease injury in other organs by mitigating the so called “organ cross talk” phenomenon. Given these promising experimental data, three phase 1b clinical trials were undertaken in healthy subjects and patients with stage 3 or 4 CKD. These studies demonstrated that FeS/SnPP were well tolerated and that they up‐regulated the cytoprotective Nrf2, ferritin, and IL‐10 pathways. Two subsequent phase 2 trials, conducted in patients undergoing ‘on‐pump’ cardiovascular surgery or in patients hospitalized with COVID 19, confirmed FeS/SnPP safety. Furthermore, interim data analyses revealed statistically significant improvements in several clinical parameters. The goals of this review are to: (i) briefly discuss the historical background of renal “preconditioning”; (ii) present the experimental data that support the concept of FeS/SnPP‐ induced organ protection; and (iii) discuss the initial results of clinical trials that suggest the potential clinical utility of an ‘oxidant preconditioning’ strategy.

## Graphical abstract

This review article describes the phenomenon of “kidney preconditioning” whereby by imposing a mild, transient oxidant stress that leads to the activation of the NRF2 cytoprotective pathway. Within 18 h, the kidney generates a host of cytoprotective proteins the confer protection against ischemic and toxic forms of acute kidney injury.

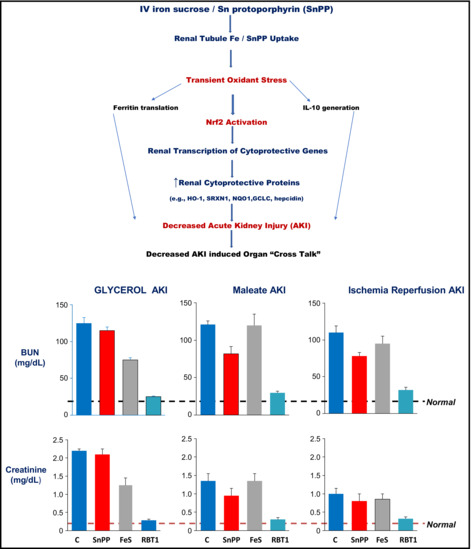

## 1 RENAL PRECONDITIONING: A BRIEF HISTORY

It has been ~100 years since W. MacNider made the seminal observation that following the induction of uranyl nitrate‐induced acute kidney injury (AKI), dogs became resistant to a second uranyl nitrate insult (WdeB, 1929). This ‘acquired cytoresistance’ was not uranyl nitrate specific given that diverse nephrotoxins (e.g., heme, HgCl_2_, gentamicin, potassium dichromate) could reproduce this result (Honda et al., 1987). Furthermore, one nephrotoxin protected against another (e.g., myoglobin‐ induced protection against HgCl_2_), a phenomenon known as ‘cross resistance’ (Honda et al., 1987). The underlying mechanism for this toxin‐induced tolerance was not ascertained. However, it was hypothesized that the first insult prevented subsequent renal toxin uptake, presumably by decreasing the expression of select renal tubular transporters. For example, multiple forms of AKI cause proximal tubular brush border megalin shedding (Venkatachalam et al., 1978), potentially limiting luminal endocytic toxin uptake. Furthermore, AKI down‐regulates multiple basolateral transporters (Sirijariyawat et al., 2019; Wang et al., 2018; Yang et al., 2021). As just some examples, maleate, cisplatin, and myohemoglobinuria have each been shown to suppress the expression of the organic cation transporter OCT2 (Table 1), the dominant determinant of proximal tubule cisplatin uptake (Wang et al., 2021). Of considerable interest, AKI‐ induced OCT2 suppression is not simply a result of tubular cell injury, per se, given that the induction of experimental azotemia in the presence of normal kidneys (induced by bilateral ureteral transection) can induce the same result (Table 1).

**TABLE 1:** Organic cation transporter 2 (OCT2) is suppressed following diverse forms of renal injury.

	Normal	Endotoxin	Glycerol	Maleate	Ischemia	Cisplatin	Ureteral transection
mRNA	1.53 ± 0.08	1.18 ± 0.12	0.55 ± 0.09	0.75 ± 0.06	1.12 ± 0.09	0.63 ± 0.09	0.74 ± 0.37
Protein	11.4 ± 0.7	8.2 ± 0.3	7.1 ± 0.6	6.5 ± 0.4	8.8 ± 0.8	3.9 ± 0.3	7.4 ± 0.4
BUN	26 ± 1	76 ± 10	166 ± 4	97 ± 12	103 ± 10	140 ± 8	130 ± 4

*Note*: OCT2 mRNA and protein levels in renal cortex following the induction of diverse forms of AKI induced in male CD‐1 mice. Challenges used were as follows: *Escherichia coli* endotoxin, 2 mg/kg; 50% glycerol, 9 ml/kg; Na maleate, 600 mg/kg; and ischemia ‐reperfusion, 25 min bilateral pedicle clamping. Assessments were made 18 h post injury induction. Cisplatin, 20 mg/kg, assessments were made 72 h post injection. To test the impact of uremia in the absence of direct renal injury, bilateral ureteral transection was induced followed by abdominal wall closure followed by renal extraction 18 h later. Normal values were determined in mice not subjected to any renal insults. The OCT2 mRNA and protein values were assessed by RT‐PCR and ELISA, respectively. OCT2 mRNA values were factored by GAPDH. OCT2 protein levels were ng/mg renal cortical protein. BUN values are mg/dl. As is apparent, each form of renal injury induced significant depressions in OCT2 gene transcription, as assessed by mRNA levels, leading to reductions in renal cortical OCT2 protein concentrations (previously unpublished data, RZ).

In 1984, our laboratory tested an alternative hypothesis: that prior renal injury causes a *direct* increase in renal tubular cell resistance to subsequent ischemic or toxic attack. To address this issue, rats were subjected to variable degrees of bilateral ischemic injury, followed by a more severe ischemic insult 24–48 h later (Zager, Baltes, et al., 1984). The first insult mitigated the injury induced by the second ischemic challenge (Zager, Baltes, et al., 1984). Hence, protection was expressed in an injury model where the issue of nephrotoxin uptake was not in play. To prove that this protection is expressed directly at the tubular cell level, proximal tubule segments were isolated from mice or rats previously subjected to diverse insults (in vivo renal or hepatic ischemia, experimental azotemia, myohemoglobinuria, partial renal ablation, ureteral obstruction‐ induced damage; Zager, 1995, 2013; Zager, Iwata, et al., 1984; Zager et al., 1995, 1999, 2014, 2016). When these isolated tubules were subjected to in vitro hypoxic, oxidant, Ca ionophore, or phospholipase A2‐ induced attack, marked cellular protection was consistently observed, (e.g., as denoted by decreased cellular lactate dehydrogenase, LDH, release). To our knowledge, these were the first demonstrations of the so‐called ‘ischemic preconditioning’ phenomenon (Zager, Baltes, et al., 1984), and that this protection is expressed directly within proximal tubule cells (Zager, 1995, 2013; Zager, Iwata, et al., 1984; Zager et al., 1995, 1999, 2014, 2016). Furthermore, that pleomorphic stressors, independent of direct tubular insults (e.g., ureteral transection‐ induced uremia; acute hepatic injury), can trigger this response underscores that direct tubular injury is not a prerequisite for the induction of this cytoresistant state. Rather, the latter appears to be part of a renal, or systemic, “stress response”.

## 2 MECHANISMS OF RENAL TUBULAR CYTORESISTANCE

A major area of investigation over the past generation has been the exploration of the biochemical, structural, and genomic changes that emerge following sublethal and lethal renal damage. This area has been extensively reviewed by others (e.g., Agarwal et al., 2016; Basile et al., 2012; Bonventre, 2007; Sharfuddin & Molitoris, 2011; Verma & Molitoris, 2015; Zuk & Bonventre, 2019) and will not be discussed at length herein. Suffice it to note that tubular injury evokes marked changes in protein, lipid, carbohydrate, epigenetic, and genomic expression, many of which likely contribute to the emergence of a secondary renal cytoprotective state. Of particular note, a plethora of studies have emphasized the importance of increased Nrf2 signaling as a major determinant of tissue resistance to damage (e.g., Leonard et al., 2006; Liu et al., 2009, 2014; Noel et al., 2015; Robledinos‐Antón et al., 2019; Vomund et al., 2017). Pioneering work by Rabb et al first identified the critical importance of Nrf2 gene signaling in renal resistance to experimental AKI (Leonard et al., 2006; Liu et al., 2009, 2014; Noel et al., 2015). Noteworthy in this regard is that oxidative stress is induced by virtually all forms of renal injury and is a key Nrf2 activator. It does so by releasing Nrf2 from the Nrf2‐Keap1 cytosolic complex, thereby permitting Nrf2 translocation to the nucleus where it binds to nuclear antioxidant elements (AREs). As a result, increased transcription of Nrf2 responsive genes results, leading to the production of a host of cytoprotective antioxidant and anti‐inflammatory proteins (e.g., heme oxygenase 1, SRXN1, GCLC, NQO1, GSTs, hemopexin, alpha 1 antitrypsin, alpha 1 microglobulin haptoglobin etc.). In concert, these cytoprotective proteins induce tissue resistance to subsequent oxidant, inflammatory, and pro‐apoptotic attack. Given this pleomorphic upregulation of diverse cytoprotective proteins, the Nrf2 pathway has been referred to as the ‘Master Regulator’ of cellular defenses (Vomund et al., 2017).

Perhaps the most extensively studied renal tubular cytoprotectant triggered by Nrf2 is heme oxygenase 1 (HO‐1). HO‐1's critical role in renal tubular cell defenses has been critically defined by pioneering work by Nath et al. (Nath, 2006, 2014; Nath & Agarwal, 2020; Nath et al., 1992), Agarwal et al. (Bolisetty et al., 2017; Hill‐Kapturczak et al., 2002; Lever et al., 2016), and others (e.g. Grunenwald et al., 2021). HO‐1's protective action has been widely ascribed to its enzymatic activity which cleaves intracellular pro‐oxidant heme, and thereby causing reciprocal increases in antioxidant biliverdin and bilirubin and generates small amounts of carbon monoxide which can trigger additional protective effects. HO‐1 also releases catalytic Fe from porphyrin rings, culminating in marked Fe driven increases in intracellular ferritin production. Because ferritin exerts multiple protective actions (Fe sequestration, ferroxidase activity, potential gene signaling), it represents a key mediator of Nrf2's and HO‐1's cytoprotective properties. Given this cascade of events, we questioned whether these pathways could be triggered by inducing mild, transient, oxidative stress by the administration of a pro‐oxidant drug, iron sucrose.

## 3 IRON SUCROSE: A TRIGGER FOR OXIDANT PRECONDITIONING AND INDUCTION OF A CYTORESISTANT STATE

**FIGURE 1:**
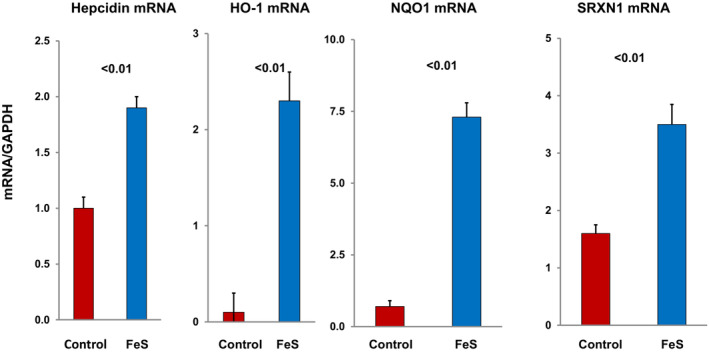
Fe sucrose (FeS)‐ induced acute up‐regulation of cytoprotective genes. Within 4 h of FeS injection, a marked transcriptional/mRNA increase of four Nrf2 sensitive cytoprotective genes (hepcidin, HO‐1, NQO1, SRXN1) was observed.

**FIGURE 2:**
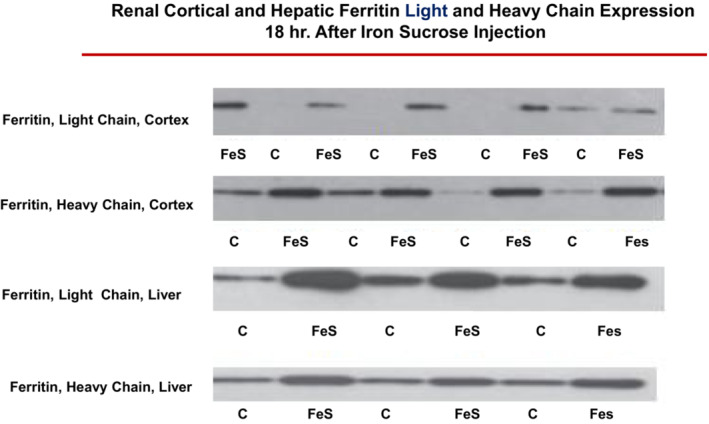
FeS induces marked increases in ferritin expression in both kidney and liver, as assessed by Western blotting. Approximately 18 h post injection of FeS, increases in heavy and light chain ferritin were observed in both kidney and liver (from Johnson et al., 2019). The latter finding has relevance to Fe‐induced hepatic cytoprotection, as described in the manuscript.

Parenteral iron sucrose (FeS) has found widespread clinical acceptance for Fe replacement therapy, most notably in chronic kidney disease (CKD) and dialysis patients. In previous studies, we explored whether FeS administration, in addition to repleting Fe stores, might increase renal tubular cell Fe content, thereby evoking a pro‐oxidant state. If so, then activation of the Nrf2 pathway and an up‐regulation of cytoprotective ferritin might result. When FeS was directly added to cultured human‐ derived proximal tubule (HK‐2) cells, marked Fe uptake was observed (Zager et al., 2004). In addition, FeS administration to mice led to renal Fe accumulation, resulting in a marked up‐regulation of multiple Nrf2 responsive genes (Figure 1) as well as ferritin (Figure 2; Johnson et al., 2019; Zager et al., 2004, 2016). As denoted by Western blotting, increases in both ferritin heavy chain and light chain components were induced (Figure 2; Johnson et al., 2019).

**FIGURE 3:**
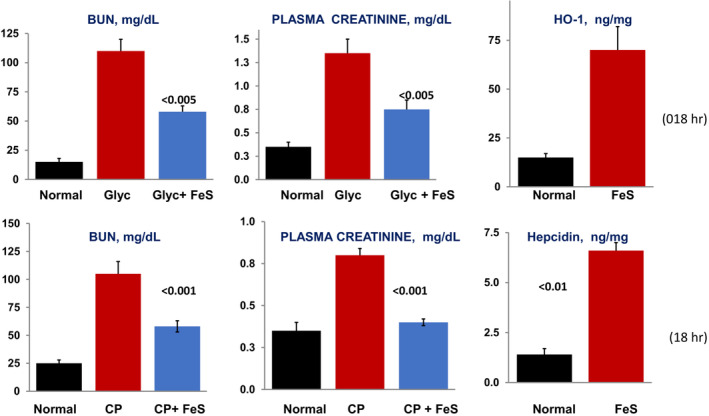
FeS‐induced protection against glycerol‐ and cisplatin‐ induced AKI (upper and lower panels, respectively). Glycerol (Glyc) AKI. Mice were preconditioned by injecting FeS, and 18 h later, glycerol AKI was induced. Saline injected mice served as controls. Glycerol induced severe AKI, as indicated by marked BUN and creatinine increases over normal values. This injury was significantly reduced in FeS preconditioned animals. The ability of FeS to up‐regulate HO‐1 protein levels in normal mice (i.e., prior to induction of glycerol AKI) is depicted in the far‐right upper panel (assessment at 18 h post FeS injection in the absence of glycerol injection). Cisplatin (CP) AKI. FeS‐preconditioned and saline‐injected control mice were injected with 20 mg/kg of cisplatin 18 h after FeS or saline pretreatment. The severity of cisplatin AKI was assessed 72 h later. Cisplatin induced severe AKI in control mice, whereas significant protection was observed in the FeS‐preconditioned mice. As shown in the bottom right panel, FeS induced a striking increase in renal cortical levels of hepcidin (a cytoprotective protein; see text), as assessed 18 h post injection into normal mice

To test whether FeS might, thus, trigger a cytoprotective state, it was administered to normal mice, and ~18 h later, they were subjected to the glycerol model of rhabdomyolysis‐ induced AKI. This AKI model is evoked, in large part, by myoglobin‐ mediated oxidative stress (Zager, 1996). As shown in the top panels of Figure 3, glycerol (Glyc) injection caused severe AKI, as denoted by marked BUN and plasma creatinine increases (Johnson et al., 2010). However, with 18 h of FeS preconditioning, substantial renal protection was observed (Johnson et al., 2010). The latter was associated with an approximate 5‐fold increase in HO‐1 protein levels (Figure 3; Johnson et al., 2010; Zager et al., 2004, 2016). However, HO‐1 activity, per se, did not appear to be directly responsible for the FeS‐ induced protection, given that this benefit was not blocked by acutely inhibiting HO‐1 activity (Sn protoporphyrin injection) at the time of glycerol injection (unpublished data; RZ). These latter findings leave open to two possibilities: *first*, HO‐1 could contribute to FeS‐ mediated cytoprotection via its non‐enzymatic actions (Dennery, 2014; Ryter, 2022; Vanella et al., 2016; Wu et al., 2021); or *second*, FeS mediated protection might arise from other Nrf2 triggered protective pathways (e.g., renal tubular ferritin or hepcidin increases, as discussed below). Dissection of this issue remains to be explored.

To further test the potential protective properties of FeS preconditioning, its ability to mitigate cisplatin nephrotoxicity was assessed. Given that significant nephrotoxicity occurs in ~30% of cisplatin‐treated patients (Li et al., 2021), FeS mediated renal preconditioning could find substantial clinical application. As shown in the bottom panels of Figure 3, within 3 days of cisplatin (CP) injection, severe AKI was observed in non‐preconditioned mice (Zager et al., 2021). However, with FeS preconditioning, a significant attenuation of AKI severity resulted. Of interest, FeS triggered a marked increase in renal cortical (as well as plasma) hepcidin concentrations (Figure 3; Zager et al., 2021). Hepcidin, a key Fe regulatory protein, has been shown to protect against experimetnal ischemic, hemoglobinuric, and sepsis‐ triggered renal damage (Scindia et al., 2015, 2019; Swaminathan, 2018). Hence, renal tubular hepcidin up‐regulation represents another potential mediator of an FeS‐induced preconditioning state.

## 4 Sn PROTOPORPHYRIN (SnPP), AN INDUCER OF THE Nrf2 CYTOPROTECTIVE PATHWAY

**FIGURE 4:**
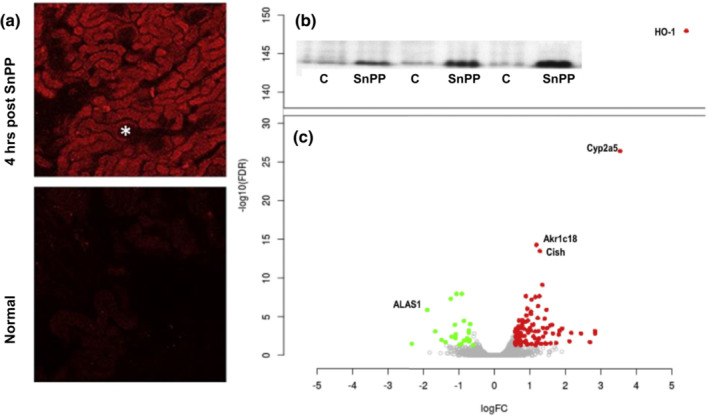
SnPP localization in proximal tubules (panel a), resulting in Nrf2 nuclear translocation (panel b) and Nrf2 activation (panel c). Panel a within 4 h of SnPP injection, SnPP's fluorescent signal was observed in mouse renal cortex, and specifically localized to proximal tubules (the asterix denotes a glomerulus, which demonstrated no uptake). The lower panel shows an absence of fluorescence in control mouse kidney tissue. Panel b an increase in Nrf2 protein is observed by Western blots in nuclear protein extracts following SnPP injection. C, control nuclear extracts. Panel c RNAseq volcano plot showing differential gene expression between kidneys exposed to SnPP × 4 h, versus saline‐injected controls. And 111 genes were found to be upregulated (red dots) and 26 genes were found to be downregulated (green dots), compared to the control group. The four genes with the highest SnPP‐induced expression were HO‐1, cytochrome p450‐2a5 (Cyp2a5), aldo‐keto reductase family 1 member C18 (Akr1c18), and cytokine inducible SH2‐containing protein (Cish). The most suppressed gene was ALAS (aminolevulinate synthase), the first enzyme controlling the heme regulatory pathway. *x* axis = log fold change (log10FC); *y* axis = false discovery rate (‐log10FDR)

**FIGURE 5:**
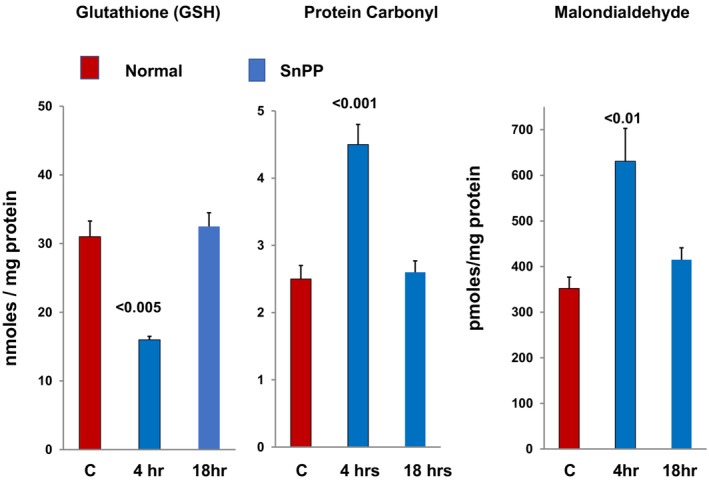
Transient oxidative stress following SnPP injection into normal mice. Within 4 h of SnPP injection, an approximate 50% decrease in renal cortical glutathione (GSH) and approximate 50% increases in renal cortical protein carbonyl and malondialdehyde levels were observed, each serving as markers of oxidative stress. The transient nature of these responses was indicated by the normalization of each of these oxidant markers within 18 h of SnPP injection.

SnPP is a well‐recognized and extensively studied HO‐1 inhibitor which blocks degradation of free heme to bilirubin. As such, it, and its analogues, have been used to prevent bilirubin formation, and hence cerebral injury, in neonates suffering from kernicterus (Stevenson & Wong, 2010). Following intravenous administration, SnPP preferentially binds to hemopexin, which despite its size (~55 kDa), is readily filtered by the glomerulus and undergoes proximal tubule endocytic uptake (Cheung et al., 2000). We have documented this process by detecting SNPP's fluorescent signal specifically within proximal tubule cells following its intravenous injection (Figure 4; Johnson et al., 2017). Following tubule uptake, SnPP induces mild, transient oxidative stress, as denoted by a decrease in renal cortical glutathione, and increases in markers of oxidative stress (protein carbonyl and malondialdehyde content; Johnson et al., 2017; Figure 5). The short‐lived nature of this response was evidenced by normalization of these three oxidant markers within *≤*18 h. A lack of renal injury was also indicated by an absence of BUN or plasma creatinine elevations following its administration (Johnson et al., 2017; Zager et al., 2016).

**FIGURE 6:**
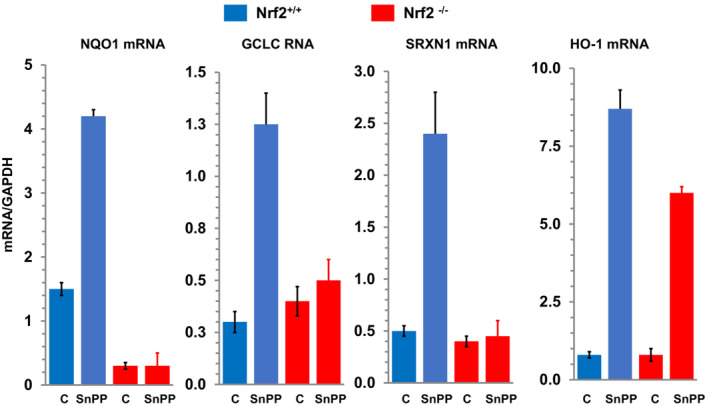
NQO1, GCLC, SRXN1, and HO‐1 mRNA responses to SnPP injection in wildtype and Nrf2^−/−^ mice. Wildtype mice demonstrated marked increases in each tested mRNA within 4 h of SnPP injection. These NQO1, GCLC, and SRXN1 responses were absent in the Nrf2^−/−^ mice. Of interest, the Nrf2^−/−^ mice manifested HO‐1 mRNA increases in response to SnPP, albeit somewhat diminished in comparison to that seen in wildtype mice. These data indicate that: (1) SnPP induces Nrf2 activation; and (2) SnPP can increase HO‐1 mRNA expression by both Nrf2 dependent and Nrf2 independent pathways.

To confirm that SnPP induced‐ oxidant stress can trigger Nrf2 activation, RNAseq analysis was undertaken in SnPP treated mice and saline injected controls. Within 4 h of administration, SnPP induced significant increases in ~20 Nrf2 sensitive genes (see Figure 4; Johnson & Zager, 2018; Johnson et al., 2017). To confirm Nrf2 activation, SnPP effects on the mRNAs of four canonical Nrf2 inducible genes (NQO1, SRXN1, GCLC, HO‐1) were assessed in wildtype mice and mice with Nrf2 deletion (Nrf2^−/−^; Johnson et al., 2017). As shown in Figure 6, SnPP induced marked mRNA increases for each assessed gene, indicating Nrf2 activation. This was confirmed by the failure of SnPP to up‐regulate NQO1, SRXN1, or GCLC mRNA in mice with Nrf2 deletion (Johnson et al., 2017). Surprisingly, the HO‐1 gene response, although somewhat diminished, was still expressed in Nrf2 deleted mice. This implies that SnPP can induce HO‐1 gene transcription via by both Nrf2 dependent, and independent, mechanisms. The nature of the latter remains unknown.

## 5 SnPP AS A CYTOPROTECTIVE AGENT

**FIGURE 7:**
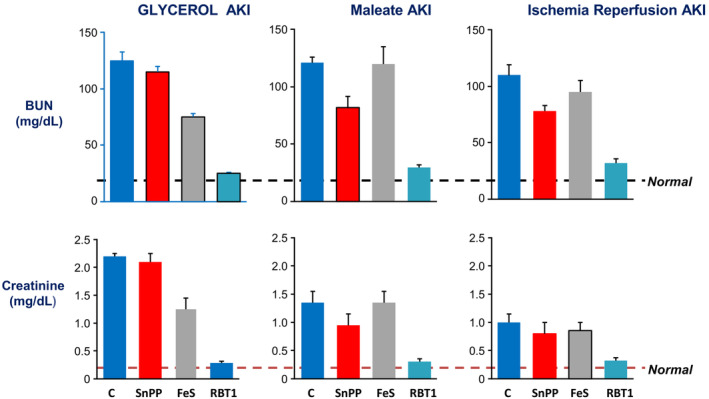
Comparative degrees of cytoprotection induced by FeS, SnPP, or FeS + SnPP preconditioning as assessed in the glycerol, maleate, and ischemia–reperfusion AKI models, conducted in CD‐1 mice. Mice were injected with either saline (controls, C), FeS alone, SnPP alone, or combined FeS + SnPP. Whereas FeS alone and SnPP alone induced modest protection, far greater protection resulted from combined FeS/SnPP preconditioning. The horizontal lines represent the mean BUN and creatinine levels in normal mice (from Johnson & Zager, 2018; Johnson et al., 2017, 2019; Zager et al., 2016; unpublished data).

Given the ability of SnPP to activate Nrf2, we tested whether it could confer protection against multiple models of experimental AKI. Toward this end, mice were administered SnPP and 18 h later they were subjected to ischemia–reperfusion, experimental rhabdomyolysis (glycerol injection), or acute maleate‐ induced nephropathy (Johnson & Zager, 2018; Johnson et al., 2017; Zager et al., 2016). In each case, a modest reduction in AKI severity was observed (Figure 7). Kaizu et al also reported that SnPP preconditioning protects against ischemic AKI (Kaizu et al., 2003). Furthermore, SnPP‐ inducible protection has been documented in multiple extra‐renal injury models, including experimental stroke (Sutherland et al., 2011), ischemic hepatic and ablative injuries (Atef et al., 2017; Pibiri et al., 2018), hyperoxic lung injury (Dennery et al., 2003), and different forms of experimental arthritis (Braza‐Boïls et al., 2011; Devesa et al., 2005; Ibáñez et al., 2011). Whether Nrf‐2 up‐regulation is directly responsible for the protection observed in each of these disease states remains unresolved. However, that SnPP‐induced protection against ischemic AKI is diminished in mice with Nrf2 deletion supports an SnPP—Nrf2 mechanistic link (Johnson & Zager, 2018).

### 5.1 The SnPP–injury paradox

There is a striking paradox concerning SnPP effects on the expression of AKI. In this regard, it has been extensively documented that if SnPP is administered *at the time of ischemic or toxic AKI induction*, a worsening of AKI severity results. This adverse effect has been attributed to HO‐1 inhibition, causing secondary decreases in cytoprotective bilirubin, biliverdin, carbon monoxide, and ferritin production (Bolisetty et al., 2017; Grunenwald et al., 2021; Hill‐Kapturczak et al., 2002; Lever et al., 2016; Nath, 2006, 2014; Nath & Agarwal, 2020; Nath et al., 1992). Conversely, and as reviewed above, if renal injury is imposed ~18–24 h post SnPP administration, renal protection is observed (Kaizu et al., 2003; Figure 7). Based on the available data, there are at least three tenable explanations for these divergent SnPP effects. *First*, although SnPP inhibits HO‐1 activity, it dramatically increases HO‐1 gene transcription, resulting in ~4‐5‐fold increases in HO‐1 protein levels (Table 2). These HO‐1 elevations can potentially increase its non‐ enzymatic protective actions (Dennery, 2014; Ryter, 2022; Vanella et al., 2016; Wu et al., 2021). *Second*, as discussed above, SnPP activates at least 20 *Nrf2 dependent* cytoprotective genes. These changes may more than compensate any negative effects arising from HO‐1 enzyme inhibition. And *third*, SnPP can activate *non Nrf2 dependent* pathways which include an up‐regulation of anti‐inflammatory IL‐10, antioxidant ferritin, improvements in mitochondrial respiration (Carr et al., 2020), and a down‐regulation of pro‐apoptotic pathways (Lang et al., 2005; Lawal et al., 2015). Hence, SnPP's preconditioning actions likely extend beyond its impacts on HO‐1 enzymatic activity, or for that matter, its activation of the Nrf2 pathway.

**TABLE 2:** Impact of iron sucrose (FeS), tin protoporphyrin (SnPP) and the combination of FeS + SnPP on the expression of cytoprotective protein gene expression.

	Normal (mRNA)	FeS (mRNA)	SnPP (mRNA)	Fe + SnPP (mRNA)	Protein# (baseline)	Protein# (18 h)
HO‐1	0.6 ± 0.05	3.5 ± 0.2	4.3 ± 0.3	4.2 ± 0.2*	15 ± 0.8	60 ± 5*
Ferritin (HC)	1.38 ± 0.01	1.49 ± 0.06	1.40 ± 0.07	1.35 ± 0.17	14 ± 2	38 ± 3*
Hepcidin	0.1 ± 0.02	0.86 ± 0.54	0.23 ± 0.09	1.1 ± 0.53*	0.8 ± 0.08	1.6 ± 0.1*
Hemopexin	0.1 ± 0.02	0.41 ± 0.2	0.56 ± 0.31	0.73 ± 0.32*	225 ± 38	330 ± 28*
Haptoglobin	0.15 ± 0.06	0.94 ± 0.31	0.19 ± 0.01	1.2 ± 0.3*	8 ± 1	55 ± 18*
α1antitrypin	0.95 ± 0.08	2.36 ± 1.10	0.73 ± 1.5	3.86 ± 0.61*	190 ± 12	285 ± 15*
α1microglobulin	0.40 ± 0.08	0.76 ± 0.13	1.03 ± 0.42	1.31 ± 0.3*	210 ± 10	242 ± 6^+^
IL‐10	0.58 ± 0.09	0.55 ± 0.29	0.29 ± 0.07	0.34 ± 0.06	300 ± 26	652 ± 35*

*Note*: Renal cortical mRNA and protein levels following iron sucrose (FeS), tin protoporphyrin (SnPP), or combination FeS + SnPP administration to male CD‐1 mice. The mRNAs were measured by competitive RT‐PCR 4 h following agent administration and are expressed as ratios to the simultaneously obtained GAPDH product, used as a housekeeping gene. The protein concentrations (#) were measured in normal mice and in mice 18 h following combination FeS + SnPP treatment using protein specific ELISAs. Ferritin levels were measured using a heavy chain specific ELISA. Values are expressed as ng/mg total renal cortical protein extract. Statistical comparisons were made between FeS + SnPP treatment versus normal mouse values. The table represents data obtained from references (Johnson & Zager, 2018; Johnson et al., 2017, 2019; Zager et al., 2016).

^+^ *p* = 0.06

* *p* < 0.001 to <0.05.

### 5.2 Assessment of a potential FeS‐SnPP synergy

Given the ability of FeS to markedly up‐regulate cytoprotective ferritin and hepcidin in renal tubules, and given SnPP's ability to activate both Nrf2 and non‐Nrf2 pathways, the potential for these two agents to exert additive or synergistic protection against AKI was tested. To this end, these two agents were administered to CD‐1 mice either alone, or in combination, and after an 18–24 preconditioning period, susceptibility to the glycerol, maleate, and the I/R models of AKI model were assessed (Johnson & Zager, 2018; Johnson et al., 2017, 2019; Zager et al., 2016; additional unpublished data). As depicted in Figure 7, FeS alone and SnPP alone each induced modest reductions in AKI severity, as assessed by BUN and plasma creatinine levels. Conversely, when the two agents were administered together, marked protection against each AKI model resulted. To explore a potential basis for this added protection, the degrees of select cytoprotective gene expression following either single or combined agent administration was sought. As shown in Table 2, at 4 h post injections, increased mRNA expression of multiple cytoprotective genes was observed. This was particularly true with combined FeS + SnPP injection, and within 18 h, marked renal cortical increases in each of the assessed cytoprotective proteins had developed (HO‐1, hepcidin, ferritin, IL‐10, hemopexin, haptoglobin, α1 antitrypsin, α1 microglobulin; Table 2). Of interest, the ferritin and IL‐10 protein increases occurred in the absence of mRNA elevations, indicating that both post transcriptional as well as transcriptional events were involved in cytoprotective protein production. Given the multitude of independent, but overlapping, FeS and SnPP effects, the exact nature of their ability to confer complementary in vivo cytoprotective actions remains to be fully defined. However, one possibility is that since SnPP inhibits HO‐1 enzymatic activity, a decrease in intracellular free iron, and hence decreased ferritin production, may result. Thus, by provision of exogenous FeS, enough free iron is made available to drive ferritin and other iron sensitive cytoprotective protein (e.g., hepcidin) production.

## 6 OXIDANT PRECONDITIONING PROTECTS AGAINST ACUTE HEPATIC INJURY

Following FeS administration, Fe is liberated and becomes bound to transferrin, thereby gaining hepatic access via the transferrin receptor. Conversely, after intravenous dosing, SnPP avidly binds to hemopexin (Morgan et al., 1988) and undergoes hepatocyte uptake via a hemopexin‐SnPP transport pathway. The high degree of hepatic SnPP uptake is underscored by prior observations that, next to kidney, the liver is the dominant site of SnPP tissue deposition (Anderson et al., 1984). We have previously observed that within 4 h of SnPP + Fe administration to mice, marked increases in hepatic cytoprotective proteins result (Zager, 2015; additional unpublished data). These findings support the concept that Fe + SnPP administration could confer protection against acute hepatic insults.

**FIGURE 8:**
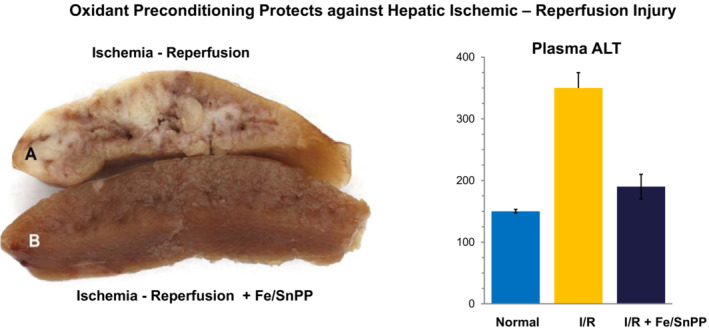
Fe/SnPP preconditioning induces hepatic protection against ischemia—Reperfusion injury. Mice were injected with SnPP + an FeS analogue (nitrated myoglobin). The latter was used to test the effect of Fe delivery without a sucrose moiety. Eighteen hours later, preconditioned and control mice were subjected to 25 min of hepatic ischemia in 3 of 5 hepatic lobes, induced by cross clamping blood supply at the portal triad. The severity of ischemic damage was assessed 18 h later by gross morphologic assessment and by measuring plasma alanine aminotransferase (ALT) concentrations. Gross ischemia‐induced necrotic injury was observed in non‐preconditioned mice (A), whereas near normal appearing hepatic tissues were seen following hepatic ischemia in preconditioned mice (B). Corresponding with these differences in gross hepatic appearance was the finding of markedly lower plasma ALT levels in preconditioned mice (derived from Zager, 2015).

Several pieces of experimental evidence support this possibility. First, previous studies from other laboratories have demonstrated that SnPP, administered as a single agent, decreases the severity of hepatic I/R injury (Atef et al., 2017) and enhances liver regeneration following partial hepatectomy (Pibiri et al., 2018). Our laboratory confirmed that preconditioning with SnPP + an Fe analogue (nitrated myoglobin) attenuates hepatic I/R injury, as well as hypertonic glycerol‐ induced liver damage (Zager, 2015; Figure 8). Thus, when these findings are considered along with the above noted SnPP‐ protective actions against stroke (Sutherland et al., 2011), hyperoxic lung injury (Atef et al., 2017), and experimental arthritis (Braza‐Boïls et al., 2011; Devesa et al., 2005; Ibáñez et al., 2011), it appears that SnPP can induce protective actions that extend beyond those expressed in kidney.

## 7 PREVENTION OF EXPERIMENTAL CARDIAC INJURY: AN EXAMPLE OF “ORGAN CROSS TALK”

**FIGURE 9:**
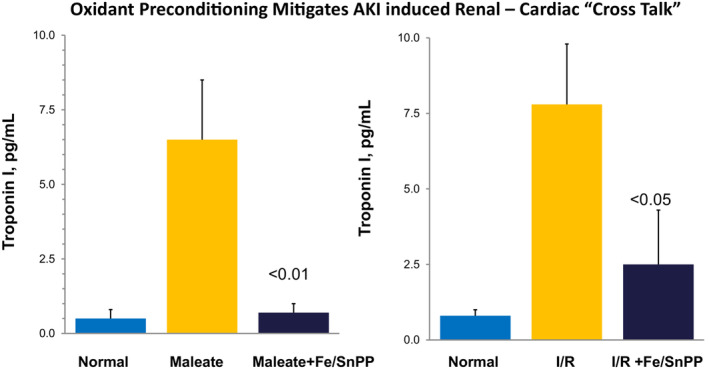
Ischemia‐ and maleate‐ AKI induce myocardial injury, as denoted by myocardial troponin I release. Both AKI models induced marked plasma troponin I increases. Preconditioning with FeS/SnPP almost totally blocked this troponin release, suggesting that by decreasing AKI severity, a decrease in ‘organ cross talk’ (kidney‐to‐ liver) results

There is compelling experimental evidence that IL‐10 administration protects against experimental myocardial infarction (Gupta et al., 2020). FeS/SnPP administration induces 2‐4‐fold increases in renal IL‐10 production and plasma IL‐10 concentrations in both mice (Zager et al., 2016) and humans (Zager & Johnson, 2020). Experimental AKI induces myocardial injury, via the phenomenon of “organ cross talk” (Rosner et al., 2012). This is a state whereby renal or extra‐renal tissue injury releases ‘damage’, or ‘inflammatory signal’, molecules (e.g., ‘DAMPs’, IL‐6) that negatively impact extra‐renal organs, such as heart (Ahuja et al., 2012; Doi & Rabb, 2016; Rosner et al., 2012; Zager & Johnson, 2020). Hence, we questioned whether FeS/SnPP‐ mediated protection against experimental AKI could potentially mitigate AKI‐ initiated myocardial damage. To this end, control mice and FeS/SnPP‐ preconditioned mice were subjected to ischemic or maleate‐ induced AKI. Associated myocardial injury was assessed by myocardial troponin I release. Within 18 h of AKI induction, marked plasma troponin I increases were observed in non‐preconditioned mice, but not in their preconditioned counterparts (Zager et al., 2016; Figure 9). Thus, these findings suggest that FeS/SnPP might confer myocardial protection by two mechanisms: (i) by stimulating IL‐10 release, decreased myocardial inflammation may result (Gupta et al., 2020); and (ii) by decreasing the severity of AKI, FeS/SnPP might decrease the release of pro‐inflammatory signals (e.g., DAMPs), thereby decreasing extra‐renal tissue damage. Whether FeS/SnPP might confer a direct myocardial cytoprotective effect, as it does in liver and kidney, remains unknown.

### 7.1 Phase 1: Clinical studies

Given the above findings of FeS/SnPP‐induced organ protection, a sequential three‐part phase 1b program (NCT04072861; NCT04072432; NCT03893799) was launched by Renibus Therapeutics, Southlake Tx, to investigate the clinical safety of their proprietary formulations of FeS, SnPP, and of a combined FeS/SnPP formulation. In addition to measurements of traditional clinical safety parameters (Johnson et al., 2019; Zager et al., 2020), potential changes in urinary AKI biomarkers (NGAL, KIM‐1, NGAL, cystatin C, albumin) were assessed. The drugs were well tolerated, the only adverse event being a mild, transient photosensitivity reaction (in ~15% of subjects; a known SnPP side effect; Johnson et al., 2019; Zager et al., 2020). Of particular note, no evidence of subclinical nephrotoxicity was observed, as evidenced by urinary biomarker assessments.

**FIGURE 10:**
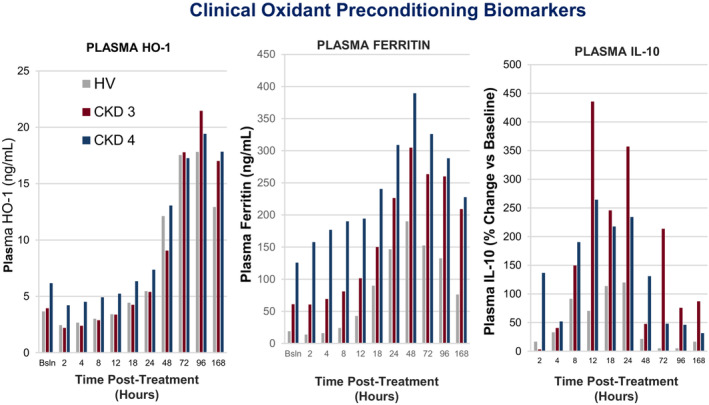
Plasma ferritin (top panel), plasma HO‐1 (middle panel), and plasma IL‐10 (lower panel) concentrations following (FeS + SnPP) treatment in healthy volunteers (HV) and subjects with either stage 3 or stage 4 CKD. Brisk and progressive ferritin and HO‐1 increases were observed over time in all assessed groups. A rapid, but more transient, increase in plasma IL‐10 was also observed, peaking at 12–48 h (unpublished data, Renibus Therapeutics).

In preclinical studies performed in mice, it was noted that the FeS/SnPP induce dose dependent increases in *renal cortical* HO‐1, ferritin, and IL‐10 which were paralleled by increases in their *plasma* concentrations (Johnson et al., 2019; Zager et al., 2016). Hence, it was posited that plasma HO‐1, ferritin, and IL‐10 levels could potentially serve as clinically useful clinical “biomarkers” of the FeS/SnPP preconditioning response. Given these observations, plasma HO‐1, ferritin, and IL‐10 levels were also assessed in the above noted phase 1b trials. As in prior mouse experiments, striking plasma HO‐1, ferritin and IL‐10 increases were observed within 24–48 h of FeS/SnPP administration (Figure 10a–c). Because FeS and SnPP were studied both alone and in combination in the phase 1b studies, it was confirmed that the FeS/SnPP‐ induced plasma ferritin increases were largely FeS dependent, whereas the HO‐1 increases primarily reflected an SnPP response. In sum, these phase 1b studies indicated that FeS/SnPP can safely activate cytoprotective pathways in humans, and by so doing, could potentially confer a clinical cytoprotective state.

### 7.2 Phase 2: Cardiovascular surgery study

Given the results of the above phase 1b studies, and the experimental evidence that FeS/SnPP can confer multi‐organ protection, Renibus launched a phase 2 trial (NCT04564833) to assess whether FeS/SnPP could induce a preconditioning biomarker response in patients undergoing “on pump” cardiovascular surgery, and whether organ protection might result. Patients were assigned to one of three groups (1:1:1) as follows: placebo (saline); low dose treatment (45 mg SnPP/240 mg FeS); or high dose treatment (90 mg SnPP/240 mg FeS). The agents were administered 24–48 h prior to surgery, allowing sufficient time for a preconditioning response to occur. Brisk plasma HO‐1, ferritin, and IL‐10 increases were observed, recapitulating the results from the phase 1b trial (Figure 10). An interim analysis was conducted on the first 60 evaluable subjects through day 30 and the results were reported to FDA. An in‐depth discussion of study design and outcomes will be forthcoming upon study completion (target of 126 subjects). However, the interim analysis suggested the following beneficial trends with Fe/SnPP preconditioning: (i) 52% and 78% reductions in AKI and major adverse kidney events by day 30 (MAKE30: e.g., dialysis, sustained renal insufficiency), respectively; (ii) reduced number of ventilator support days and ICU stays (*p* < 0.05); (iii) a 2 day reduction in total hospitalization days; and (iv) a statistically significant reduction in 30‐day hospital readmission rates; *p* = 0.003. As in the phase 1 study, the only treatment‐related adverse event was mild transient photosensitivity, again seen in ~15% of subjects. Whether the observed non‐renal endpoints reflected direct Fe/SnPP effects on extrarenal organs or were potentially secondary to protective effects of elevated plasma ferritin, IL‐10, or HO‐1 concentrations remains to be defined. Furthermore, all of the above observations require confirmation in a larger study. To this end, a phase 3 trial is pending, having received FDA fast track approval.

### 7.3 Phase 2: COVID 19 study

Given the high incidence of AKI and multi‐organ complications in hospitalized COVID 19 patients, a randomized, placebo‐controlled study (NCT04364763) was undertaken to evaluate potential benefits of single agent SnPP treatment. Disease status was assessed using the 8‐point WHO Ordinal Scale (López‐Medina et al., 2021), and by the length of hospitalization. The study was terminated early due to enrollment difficulties (a result of many competing COVID related trials). However, data from 43 hospitalized subjects (1:1 SnPP: placebo enrollment) were analyzed. Within 7 days of SnPP treatment, a 1.5‐point reduction in the 8‐point WHO Ordinal Scale was observed *p* = 0.0163. This corresponded with a 68.1% decrease in hospital length of stay; *p* = 0.0354. The incidence of AKI in both control and SnPP‐treated patients were not sufficient to permit a meaningful comparative analysis. Again, the only treatment‐related adverse event was photosensitivity, as noted in the above studies. Hence, these findings further support SnPP safety and potential efficacy in disease mitigation in critically ill patients.

## 8 CONCLUSIONS

The concept of ‘organ preconditioning’ dates back almost 100 years, but no proven method to effectively trigger it and its associated organ protection have emerged. Within the past 25 years, the organ protective actions of the Nrf2 pathway have been revealed, and its potential to mitigate experimental AKI has become clear. Recent work has demonstrated that FeS and SnPP, used either alone, or more effectively in combination, can activate Nrf2 and additional cytoprotective pathways (e.g., IL‐10, ferritin). As a result, marked renal, and potentially extra‐renal, organ protection against diverse forms of experimental tissue injury can result. Recent phase 1b / phase 2 studies suggest both the safety and potential clinical utility of this Fe/SnPP ‘oxidant preconditioning’ strategy. However, it is clear that the current findings require confirmation in a planned phase 3 trial.

## FUNDING INFORMATION

This work was supported by research grants from the National Institutes of Health (DK38532), discretionary research funds from Fred Hutchinson Cancer Center, Seattle, WA, and by Renibus Therapeutics, Southlake Tx.

## CONFLICT OF INTEREST

Since the start of clinical trials, the author has been a paid consultant for Renibus Therapeutics, Southlake, TX.

## ETHICS STATEMENT

All animal experiments were conducted with IACUC approval. The clinical studies were all approved by IRBs and signed consents were obtained in all circumstances.
